# A Nanomechanical Analysis of Deformation Characteristics of 6H-SiC Using an Indenter and Abrasives in Different Fixed Methods

**DOI:** 10.3390/mi10050332

**Published:** 2019-05-18

**Authors:** Jisheng Pan, Qiusheng Yan, Weihua Li, Xiaowei Zhang

**Affiliations:** 1 School of Electromechanical Engineering, Guangdong University of Technology, Guangzhou 510006, China; qsyan@gdut.edu.cn (Q.Y.); zxwei@gdut.edu.cn (X.Z.); 2 Guangdong Nanogrind Technology Ltd., Foshan 528225, China; 3 Mechanical, Materials, Mechatronic and Biomedical Engineering, University of Wollongong, Wollongong, NSW 2522, Australia; weihuali@uow.edu.au

**Keywords:** 6H-SiC, indentation, deformation, material removal mechanisms, critical load

## Abstract

The super-precise theory for machining single crystal SiC substrates with abrasives needs to be improved for its chemical stability, extremely hard and brittle. A Berkovich indenter was used to carry out a systematic static stiffness indentation experiments on single crystal 6H-SiC substrates, and then these substrates were machined by utilizing fixed, free, and semi-fixed abrasives, and the nanomechanical characteristics and material removal mechanisms using abrasives in different fixed methods were analyzed theoretically. The results indicated that the hardness of C faces and Si faces of single crystal 6H-SiC under 500 mN load were 38.596 Gpa and 36.246 Gpa respectively, and their elastic moduli were 563.019 Gpa and 524.839 Gpa, respectively. Moreover, the theoretical critical loads for the plastic transition and brittle fracture of C face of single crystal 6H-SiC were 1.941 mN and 366.8 mN, while those of Si face were 1.77 mN and 488.67 mN, respectively. The 6H-SiC materials were removed by pure brittle rolling under three-body friction with free abrasives, and the process parameters determined the material removal modes of 6H-SiC substrates by grinding with fixed abrasives, nevertheless, the materials were removed under full elastic-plastic deformation in cluster magnetorheological finishing with semi-fixed abrasives.

## 1. Introduction

Single crystal SiC is a third-generation semiconductor which performs well in terms of high breakdown electric field, high thermal conductivity, large band gap, high saturated electron drift velocity, low dielectric constant and good resistance to radiation damage, all of which open up completely new classes of commercial and military applications which are currently impossible or unaffordable with silicon or gallium arsenide [1,2]. The most widely used examples include their use as sources of white light for optical storage, as displays, and in radar and communication technology, and in automotive and oil exploration industries and in high-radiation environments [3,4]. Of the many different silicon carbide polytypes, 3C-SiC, 4H-SiC and 6H-SiC are the most commonly applied. Depending on the particular application, the requirements for the resulting surface of SiC substrates are stringent and often stipulate that the roughness (Ra) must be less than 0.3 nm, the wafers must have flat, smooth, and damage free surfaces; this means the machined quality directly determines the applied value and performance of the device [5,6]. However, the high hardness, stiffness, and strength of single crystal SiC make it very difficult to obtain a high-quality surface finish by mechanical methods, even with a diamond cutting tool [7].

In recent years some researchers have become interested in analyzing the mechanical properties of single crystal SiC; Yin et al. used Vickers indention and nanoindentation to carry out indentation experiments on the 6H-SiC substrates, and used grinding and polishing tests to investigate microfracture, residual damage, and surface roughness associated with material removal and surface generation [8]. Meng et al. performed a nanoscratching test on 6H–SiC with a Berkovich diamond indenter and found that the deformation and removal of 6H-SiC while nanoscratching with a sharp indenter was completely different from the single-point diamond turning (SPDT) method [9]. Goel et al. carried out a diamond turning test on single crystal 6H-SiC at a cutting speed of 1 m/s on a precision diamond turning machine to elucidate the microscopic origin of ductile-regime machining, and obtained a surface finish of Ra = 9.2 nm [10]. Yan et al. used a Berkovich nanoindenter to investigate the subsurface damage of SiC in nanoindentation tests, and found that the depth of subsurface damage was much larger than that in indentation tests, and the damaging mechanism of SiC was completely different from single crystalline silicon [11]. Xiao et al., carried out molecular dynamics (MD) simulations to investigate the atomic scale details of ductile deformation while machining of 6H SiC, and found that a taper cutting experiment on a single crystal 6H SiC wafer produced a ductile-cut surface. Moreover, a micro Raman spectroscopy of the machined surface revealed no peaks for amorphous SiC, which agreed with the MD result [12]. Lee et al. proposed a hybrid polishing technique using a mixed abrasive slurry (MAS) with colloidal silica and nanodiamonds to investigate the hybrid removal mechanism of MAS on SiC [13]. Goel et al. carried out diamond turning of single crystal 6H-SiC at a cutting speed of 1 m/s on a precision diamond turning machine to elucidate the microscopic origin of ductile-regime machining; a surface finish of Ra = 9.2 nm was obtained and a brittle–ductile transition was observed [14]. Li et al. carried out the nanoindentation test for 6H-SiC with a Berkovich indenter and set up the three-dimensional finite element simulation, the plastic deformation and cracks morphology and mechanical properties were analyzed with the maximum load P (max) [15]. Nawaz et al. investigated the nanoscale elastic-plastic deformation behavior of single crystal 6H-SiC systematically by using nanoindentation with a Berkovich indenter and observed the effect of loading rates on the critical pop-in load, pop-in displacement and maximum shear stress [16]. Pang et al. presented an experimental and numerical analysis of the deformation behavior of single-crystal 6H-SiC in nanoindentation, the results showed that classical crystal plasticity theory can be reliably applied in predicting plastic deformation of ceramic at small scales [17]. Lu et al. described the mechanical planarization machining of SiC substrates involving the Si face and C face of N-type 4H-SiC, N-type 6H-SiC, and V-type 6H-SiC with a sol-gel polishing pad, the removal mechanism of SiC substrates was investigated by nanoindentation and nanoscratching [18].

While these studies did not present concrete nanomechanical data and generally used the mechanical data in the literature to analyze material removal mechanism, nor did they analyze the material removal mechanism of single crystal SiC from a mechanical perspective whilst using abrasives in different fixed methods. In this paper, a nanomechanical test system was used to test the nanoindentation of single crystal 6H-SiC materials to obtain the hardness, modulus and loads for elastic-plastic and plastic-brittle transitions of this material. The single crystal 6H-SiC materials produced by the same factory were machined by rotational grinding with fixed abrasives, lapping with free abrasives and magnetorheological (MR) finishing of semi-fixed abrasives, respectively. Then, the mechanical properties of the materials in brittle-plastic transition under the three fixed methods of abrasives with optimal experimental conditions were calculated. On this basis, this study revealed the mechanical behavior and material removal mechanisms of single crystal 6H-SiC under the effects of abrasives by combining the morphologies of the machined surfaces and the results of nanoindentation experiments.

## 2. Experimental

### 2.1. Materials

The 6H n-type SiC Dummy Grade wafers (the C face and Si face can be identified by the primary orient flat and secondary orient flat) purchased from tankeBlue Semiconductor Co. Ltd., Beijing, China were used in this experiment. Indentation and nanoscratching tests were performed on the polished wafers and grinding and lapping experiments were carried out on the as-cut wafers and double side lapped wafers. The wafers are 300 ± 10 µm thick, the surface roughness Ra of these as-cut wafers, double side lapped wafers, and polished wafers are about 0.21 μm, 73 nm, and 0.3 nm, respectively.

### 2.2. Nanoindentation Experiment

Nanoindentation was carried out by utilizing the Agilent G200 Nanoindenter system (Santa Clara, CA, USA); it can also be used for nanoindentation and nanoscratch, and works as a nanomechanical microscope. This experimental process was carried out automatically, according to a set of procedures to improve the reliability and competition of the experimental data. Within a total displacement range of 1.5 mm, the indenter can generate indentations deeper than 500 µm with a displacement resolution of less than 0.01 nm and a load resolution of 50 nN, respectively, under maximum load (standard) higher than 500 mN.

In nanoindentation experiments, single crystal SiC substrates were fixed onto the carrier plate using hot melt adhesives. The authors conducted a series of static stiffness experiments on Si and C faces of 6H-SiC under loads increasing from 1 mN to 500 mN using a triangular pyramid Berkovich indenter produced by the Agilent Company (Santa Clara, CA, USA). The edge plane and edge of the indenter showed 65.3° and 77.05° angles with the center line, and the equivalent cone angle was 70.32°. In this experiment, nine points were tested under each load at 3 a.m. to avoid any influences from the surrounding environment; after which the hardness *H* and the elastic module *E* of the points with maximum depth under static stiffness were obtained directly from the software and their average values were calculated as experimental results after excluding particular data. 

### 2.3. Fixed Abrasive Machining

**Figure 1:**
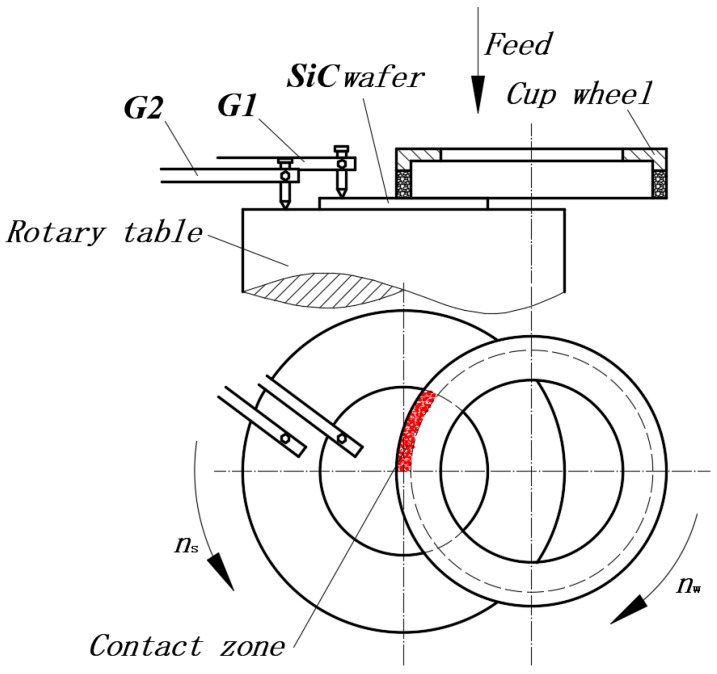
Principle of workpiece rotation grinding.

The grinding experiments using fixed grinding abrasives were carried out on the DMG-6011V vertical, uniaxial and high-precision end face grinder manufactured by the Lapmaster SFT Corp. (Tokyo, Japan). On this machine tool, cup-shaped grinding wheels were used to cut and grind the workpieces axially. The contact length, contact area, and cutting angle of the grinding wheels and workpieces were constant and half of the machined workpieces were always outside the grinding wheels. Furthermore, by using online thickness measuring devices, crystal plates were ground precisely and were not affected by any wear in the grinding wheels (Figure 1). The movement of the spindle of this machine tool was set at 0.01 μm per unit for its minimum rate of movement is 0.01 μm/sec, while the grinding thickness was controlled at 0.1 μm. The experiments utilized #325 metal-bonded and #8000 ceramic-bonded diamond grinding wheels, with deionized water as a coolant. The experimental parameters are shown in Table 1.

### 2.4. Free Abrasive Machining

The lapping experiments using free abrasives were carried out on the KD15BX (Dongguan KIZI Precision Lapping Mechanical Manufacture Co., LTD., Dongguan, China) precision plane grinder. This machine includes precision lapping plates, a trim ring, ceramic plates, cushions, clump weights, a retaining hook structure, a precision dressing machine, and a supply system of slurry. After being dressed for flatness, the lapping plates had a total thickness variation (TTV) of less than 10 μm and the parallelism of the upper and lower sides of the ceramic plates was less than 2 μm. The workpiece was fixed onto the ceramic plates with paraffin and faced to the lapping plate, while the trim ring was fixed outside the round ceramic plates. Clump weights were placed over and isolated with the ceramic plates by cushions. The pulley of the retaining hook structure was tangential with the outer cylinder of the trim ring so that the trim ring and ceramic plates could rotate due to friction between the retaining hook structure and the lapping plates. The experiments were carried out on the cast iron lapping plate and ceramic lapping plates using W14 and W1.5 diamond abrasives for 5 min as the lapping plates were rotating at 80 rpm. The mass fraction of abrasives in the slurry, flows of slurry, and lapping pressure were 4 wt.%, 15 mL/min, and 30 kPa, respectively.

### 2.5. Semi-fixed Abrasive Machining

**Figure 2:**
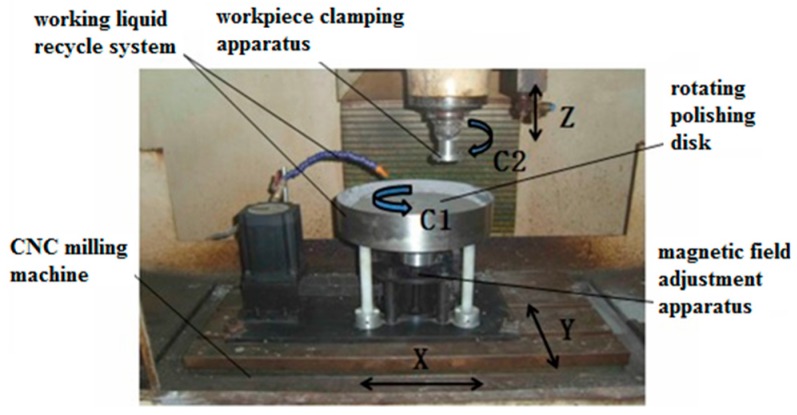
Experimental apparatus for cluster MR effect in plane polishing.

The MR finishing experiments with semi-fixed abrasives were carried out using the experimental equipment for MR plane finishing that was developed by the laboratory (Figure 2). This equipment utilized the servo motion of CNC milling machines and was controlled in the four directions (X, Y, C1 and C2) needed for the polishing process via programming. To analyze the material removal mechanism of single crystal SiC substrates under MR processing, N35 cylindrical permanent magnets (20 mm × 15 mm) with a flat bottom were arranged circumferentially on the polishing plates in a 135 mm diameter in the same direction as the magnetic poles to form a cupped polishing circle. The single crystal 6H-SiC substrates were 2 inches in diameter and were faced directly in the center of the magnetic pole by adjusting movement in the X and Y directions. The distance between the lower surface of the substrates and the upper surface of the polishing plates was set at 0.8 mm by controlling the Z axis. The workpiece was static, while the polishing plates rotated at C1 = 200 r/min to machine the workpiece for 35 min, thus producing arc polishing belts on the workpiece. In the experiment, water-based MR fluids mixed with certain proportions of abrasives were used as MR polishing fluids. These MR polishing fluids consisted mainly of W3.5 carbonyl iron powders (4 wt.%), deionized water (88 wt.%), W3.5 diamond powders (4 wt.%), and stabilizer (4 wt.%).

The surface roughness was measured with a MarWin XT20 (Mahr, Goettingen, Germany) surface roughometer, and the surface morphology was analyzed by OLS4000 (Olympus Corporation, Tokoy, Japan) laser confocal microscopy and a scanning electron microscope (SEM) (Hitachi, Ltd., Tokoy, Japan).

## 3. Results and Discussion

### 3.1. Results and Theoretical Analysis of this Nanoindentation Experiment

**Figure 3:**
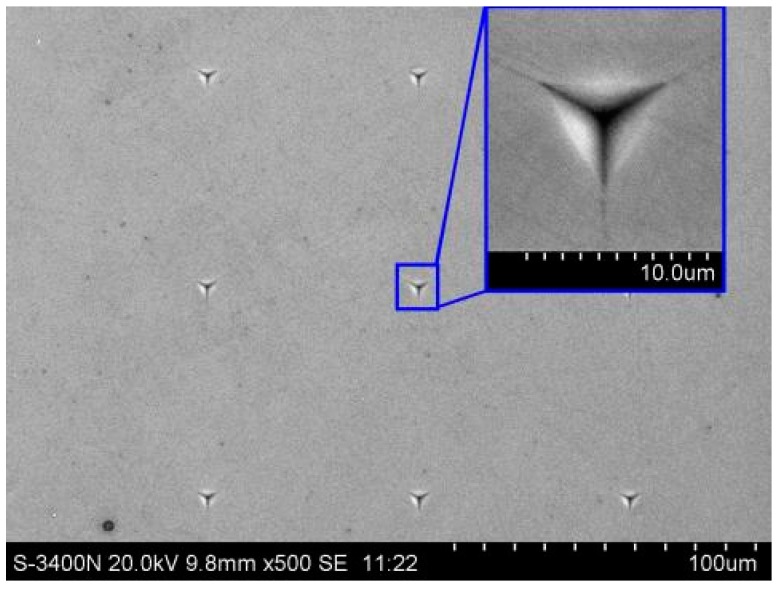
Surface topography of nanoindentation.

The experiments were carried out on the C and Si faces of polished 6H-SiC substrates by utilizing the static stiffness method under 1 mN, 2 mN, 5 mN, 10 mN, 20 mN, 50 mN, 100 mN, 200 mN, 300 mN and 500 mN loads. Each experiment was repeated nine times in a 3 × 3 array. The surface morphologies of the 6H-SiC substrates obtained from the experiment are shown in Figure 3 and the hardness and elastic moduli of the C and Si faces are shown in Table 2.

The results indicate that single crystal SiC substrates showed the obvious effects of indentation size, whilst due to the influences of surface roughness, oxide layers on the surfaces and elastic-plastic deformation of the workpieces, the hardness and elastic modulus gradually increased as the loads and depth of indentation increased in a certain range. Afterwards, due to the soft base of the hot melt adhesives used as test patches, the hardness and elastic modulus gradually decreased as the loads and depth of indentation increased. The hardness of static stiffness of C and Si faces under 500 mN loads were 38.596 Gpa and 36.246 Gpa, respectively, which were basically consistent with the experimental result (38 Gpa) of Chen et al. [19]. Moreover, the elastic moduli of the static stiffness of C and Si faces under 500 mN loads were 563.019 Gpa and 524.839 Gpa, respectively, which were slightly larger than the result (448 Gpa) of Mehregany et al. [20]; this was probably due to the soft base of the hot melt adhesives. Therefore, the C and Si faces of single crystal 6H-SiC showed slightly different mechanical properties and demonstrated obvious anisotropy. Furthermore, the hardness and elastic moduli of the C face were larger than the Si face, which indicated that the C face was more difficult to machine than the Si face.

**Figure 4:**
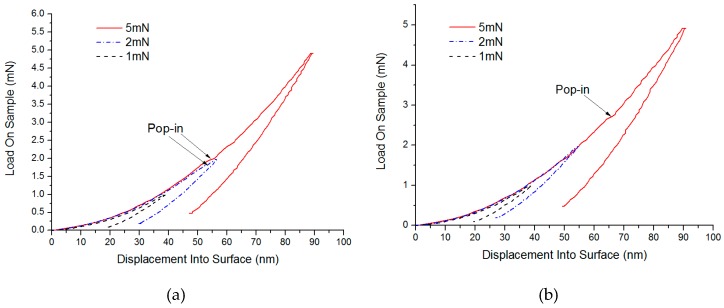
Test results of Single crystal 6H-SiC substrates under small loads (**a**) Load and depth curve in small loads of C face and (**b**) Load and depth curve in small loads of Si face.

**Figure 5:**
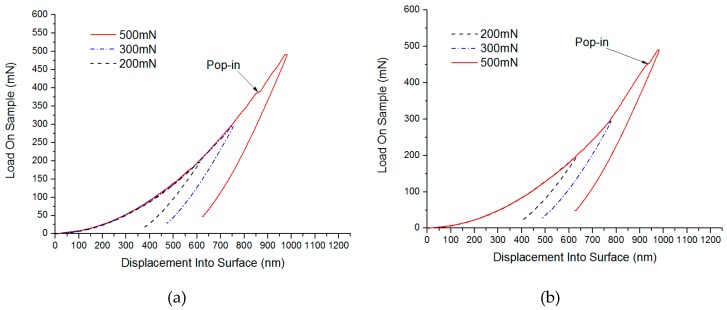
Test results of Single crystal 6H-SiC substrates under large loads (**a**) Load and depth curve in large loads of C face and (**b**) Load and depth curve in large loads of Si face.

By using the static stiffness method, the load-depth curves between loads and indentation depth of the indenter are shown in Figure 4 and Figure 5. Here, at 1.941 mN, a slight pop-in appeared on the C surface of SiC (considering the effects of the soft base of hot melt adhesives, the indentation depth was not used as a reference standard), while a micro pop-in was found on Si surface of 2.710 mN. Therefore, pop-ins under small loads were considered to be the turning point where materials changed from elastic to plastic deformation. Similarly, under higher loads, micro pop-ins appeared on the C and Si surfaces of SiC in 385.362 mN and 448.217 mN, respectively, showing that obvious brittle fractures occurred to the SiC materials under these loads.

In the nanoindentation experiment, a single ideal diamond grinding grain acted perpendicularly onto the single crystal SiC substrates. For brittle materials, as the load increased, they went through stages such as elastic and plastic deformation, and brittle fracture. Elastic deformation was mainly shown as an increase or decrease of the interatomic distance where elastic recovery occurred after unloading, while plastically deformed materials cannot recover to their original shapes after unloading. Under the effect of plastic deformation, plastic flows and plastic budges were generated on the materials which caused the easy formation and accumulation of defects such as fault and slippage, and because these stresses were beyond the minimum stress required by crack extension, brittle fractures caused the materials to produce microcracks which travelled inside the materials. The transition from elastic to plastic deformation and then to brittle fracture was attributed to the pop-ins of critical stress and strain on the materials. Therefore, the effective evaluation and test of the critical stress and strain of the materials provided an important basis for analyzing the material removed. 

According to the simple contact load model proposed by Stevanovic for predicting elastic, elastic-plastic, and plastic deformations [21]:

(1) $$F_{o}=\frac{9\pi^{3}}{16}H^{3}{\left(\frac{\rho }{E}\right)}^{2}.$$

When the force on surfaces of the material is $F\leq \frac{1}{15}F_{o}$, it is considered that pure elastic deformation occurred, and elastic-plastic deformation occurs when the force is $\frac{1}{15}F_{o}<F<15F_{o}$; moreover, pure plastic deformation appears when the force is $F\geq 15F_{o}$. In the Equation, H, ρ, and *E* represent the hardness of the materials, the radius of curvature of grinding grains on the materials, and the elastic moduli of the materials, respectively.

In Equation (1), the hardness and elastic moduli of single crystal SiC were obtained through the nanoindentation experiment, while the radius ρ of the curvature of the Berkovich indenter was unknown. When $F=15F_{o}$ was used as the critical load for the plastic transition of single crystal 6H-SiC, then

(2) $$F_{C}=15\frac{9\pi^{3}}{16}38.5^{3}{\left(\frac{\rho_{C}}{560}\right)}^{2}=1.941\;\mathrm{mN}$$

(3) $$F_{Si}=15\frac{9\pi^{3}}{16}36^{3}{\left(\frac{\rho_{Si}}{524}\right)}^{2}=2.71\;\mathrm{mN}.$$

By solving Equations (2) and (3), $\rho_{C}=0.2\;\mathrm{mm}$ and $\rho_{Si}=0.3\;\mathrm{mm}$. As a single crystal SiC is very hard, when the Berkovich indenter was pressed into the surfaces of carbon and silicon in batches, it was worn, which further influenced the precision of the measuring results. The silicon surface was trimmed and calculated according to $\rho_{C}$ value, so theoretically the critical load for the plastic transition of the silicon surface under the same conditions was

(4) $$F_{Si’}=15\frac{9\pi^{3}}{16}36^{3}{\left(\frac{0.2}{524}\right)}^{2}=1.77\;\mathrm{mN}.$$

Therefore, when the forces on the surfaces of single crystal 6H-SiC materials were $F_{c}\leq 8.63\;\mu N$ and $F_{si}\leq 7.87\;\mu N$, pure elastic deformation occurred, but if the forces were $8.63\;\mu N<F_{c}<1.941\;\mathrm{mN}$ and $7.87\;\mu N<F_{si}<1.77\;\mathrm{mN}$, elastic-plastic deformation appeared, and if the forces were $F_{C}\geq 1.941\;\mathrm{mN}$ and $F_{si}\geq 1.77\;\mathrm{mN}$, pure plastic deformation occurred.

When the materials were processed by plastic removal, plastic flows which did not result in cracks were observed on the surface layers, but because the materials hindered the dislocation guide, while the loads increased constantly, many dislocations accumulated at a point to form a dislocation pile-up group. Once the force reached a certain limited value, the dislocation pile-up group induced the generation and extension of cracks, which turned into brittle removal [22], and therefore the critical loads needed for the materials to produce cracks were those for the brittle transition of materials, and the critical loads were mainly determined by mechanical properties of the materials. These mechanical properties included the hardness H, the fracture toughness Kc, and elastic module E of the materials. Based on the model of indentation fracture mechanics [23,24], the critical load P* for brittle materials changing from plastic deformation to brittle fracture was expressed as

(5) $$P^{*}=54.5(\alpha /\eta^{2}\gamma^{4})(K_{c}{}^{4}/H^{3})$$

where α (for ordinary Victorinox indenter, α = 2/π), *η* and *γ* (*η* ≈ 0.6, *γ* ≈ 0.1) are constants. *K_c_* and *H* represent fracture toughness (generally, *K_c_* is 1.9 Mpa m1/2 for single crystal SiC [25]) and hardness of materials. The theoretical critical loads for the brittle fractures of the C and Si faces of single crystal SiC are defined below respectively.

(6) $$P^{*}{}_{C}=54.5((2/\pi )/(0.6^{2}0.1^{4}))(1.9^{4}/38.5^{3})=366.82\;\mathrm{mN}$$

(7) $$P^{*}{}_{\mathrm{Si}}=54.5((2/\pi )/(0.6^{2}0.1^{4}))(1.9^{4}/36^{3})=448.67\;\mathrm{mN}.$$

Note that the results of this theoretical calculation were consistent with those obtained in the nanoindentation experiment and can be used as basic parameters for analyzing the removal of materials machined by different fixed abrasives.

### 3.2. The Results of Grinding with Fixed Abrasives and Materials Removal Analysis

**Figure 6:**
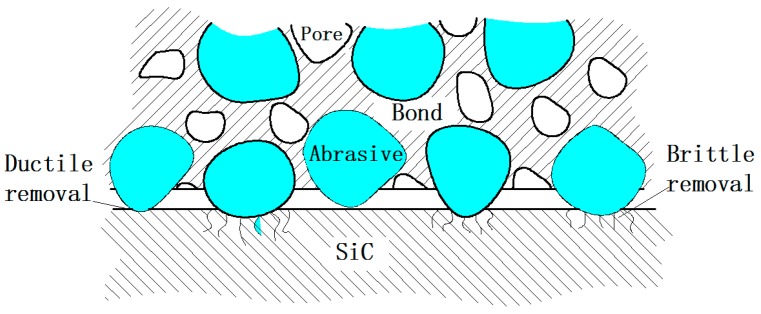
Schematic diagram of fixation abrasive grinding.

**Figure 7:**
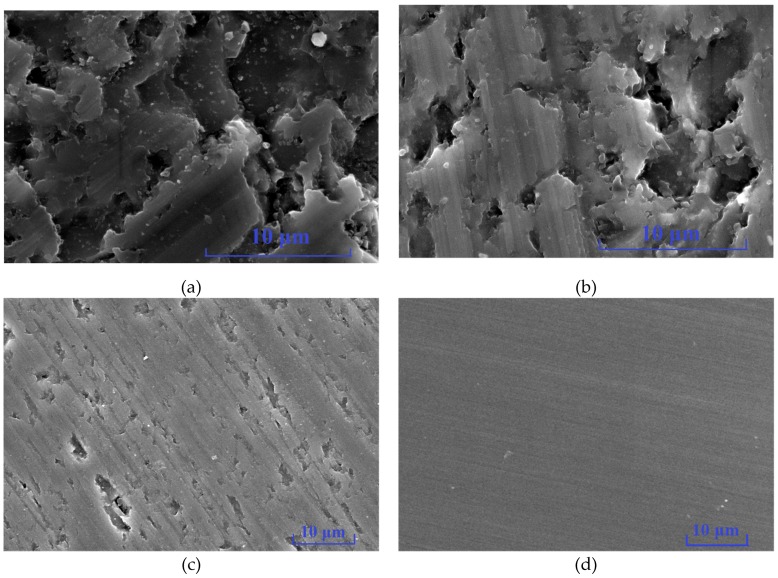
Morphology of Single crystal 6H-SiC substrates ground with different grades of grit (**a**) No.1 (Ra 0.303 μm), (**b**) No.2 (Ra 0.029 μm), (**c**) No.3 (Ra 0.015 μm) and (**d**) No.4 (Ra 0.002 μm).

Since the grains in the grinding experiment were always fixed onto the grinding wheels, it is known as machining with fixed grinding grains (Figure 6). Figure 7 presents the surface SEM morphologies of ground 6H-SiC substrates. Note also that the diameter, feed, and linear speed of the grinding wheels influences the surface finish, so the morphologies of the machined surfaces obviously changed and the methods for removing material were also different. Under the No.1 process condition, most single crystal SiC was removed through brittle fracture and small amounts of plastic removal. After decreasing the feed of the grinding wheels in the No.2 process, brittle removal dominated and there was more plastic removal. As process No.3 shows, the linear speed of the grinding wheels affected the material removal modes, and the faster the linear speed, the larger the proportion of plastic removal. Although same process parameters were used in the No.3 and No.4 conditions, No.4 resulted in completely different effects where materials were mainly removed via the plastic mode because the grains in the grinding wheels had different diameters and densities.

Many studies have shown that the amount of material removed by grinding is related to the cutting depth $d_{c}$ of the grit, which in turn is related to the properties of materials and the maximum thickness of undeformed substrate $h_{\max}$. When the maximum thickness of undeformed substrate $h_{\max}$ is less than the critical grit cutting depth $d_{c}$, the plastic domain of grinding is achieved [26].

When grinding takes place the brittleness $H/K_{c}$ of materials is the principal factor influencing the brittle-plastic removal of materials.

Bifano et al. [26] put forward the critical grit cutting depth $d_{c}$ for the brittle-plastic transition of material removal modes in grinding.

(8) $$d_{c}=0.15\left(\frac{E}{H}\right){\left(\frac{K_{c}}{H}\right)}^{2}.$$

In Equation (9), *E*, *H*, and *K_c_* stand for the elastic module, hardness, and fracture roughness of materials, respectively, so by substituting the experimental results above into Equation (8), the authors found that the critical grit cutting depth for C and Si faces of single crystal 6H-SiC were 5.3 nm and 6.1 nm, respectively.

During grinding, the maximum thickness of undeformed substrate $h_{\max}$ denotes the maximum depth of grinding grit cutting into the workpiece. Apart from the size of the grit and the feed rate of the grinding wheels passing over the workpiece, the maximum thickness of undeformed substrate was also related to factors such as the speed at which the workpieces and grinding wheels rotate and the size of grinding wheels. During grinding realized by the self-rotation of the workpieces, suppose that the speed at which the grinding wheel and workpieces rotate, and the feed and radius of the grinding wheels are represented by $n_{s}$, $n_{w}$, *f* and *R*, respectively. $L_{w}$ and *W* stand for the circumference and tooth width of layers of grinding material of the cupped grinding wheels. Furthermore, *C*, F_v_ (when the diamond density was 3.25 $g/{\mathrm{cm}}^{3}$, $F_{v}=0.25C$) and $r_{m}$ indicate the concentration of grinding materials of diamond grinding wheels, the volume fraction of the layers of material in the grinding wheels, and the radius at any point on the surface of the workpieces, respectively. According to the study by Shang [27], the maximum thickness of undeformed substrate $h_{\max}$ was:

(9) $$h_{\max}=2.239R{\left(\frac{4fr_{m}n_{w}}{L_{w}WCn_{2}^{2}}\right)}^{0.4}.$$

In this experiment, the radius *R* of the grinding wheels and the tooth width *W* of the layers of grinding material in the cupped grinding wheels were 100 mm and 3 mm, respectively. The $r_{m}$ value is the position of the edges of the workpiece (for a 2-inch diameter workpiece, r_m_ was 25.4 mm). Moreover, the value of concentration *C* in Equation (9) can be obtained through simple geometric relationships, as shown in the following equation [28]:

(10) $$C=\frac{4f}{d_{g}^{2}{\left(\frac{4\pi }{3v_{d}}\right)}^{2/3}}.$$

In Equation (10), $d_{g}$, *v_d_*, and *f* represent the equivalent spherical diameter of diamond particles, the volume fraction of diamonds in the grinding wheel, and the fraction of diamond particles that actively cut when grinding, respectively. The grinding wheel used in the present study had a density of 100, or in other words, the volume fraction *v_d_* was 0.25. To obtain the value of C, it was assumed that only one-half of the diamond particles on the wheel surface was actively engaged in cutting [28], or the value of *f* was equal to 0.5.

By substituting the experimental conditions and parameters in Table 1 into Equations (9) and (10), the maximum thicknesses of undeformed substrates from processes No.1 to No.4 were 47.59 nm, 9.95 nm, 7.56 nm, and 0.43 nm, respectively. It is obvious that the maximum thicknesses of undeformed substrates from No.1 to No.3 were larger than the critical grit cutting depth dc, and therefore the materials were removed by brittle fractures. As the maximum thickness of undeformed substrates decreased the proportion of plastic removal on the surfaces gradually increased, however, the maximum thickness of the undeformed substrate in process No.4 was smaller than the critical grit cutting depth dc, so the material removal modes were completely transformed into plastic flows.

### 3.3. The Lapping Results of Free Abrasives and Analysis of Material Removal

**Figure 8:**
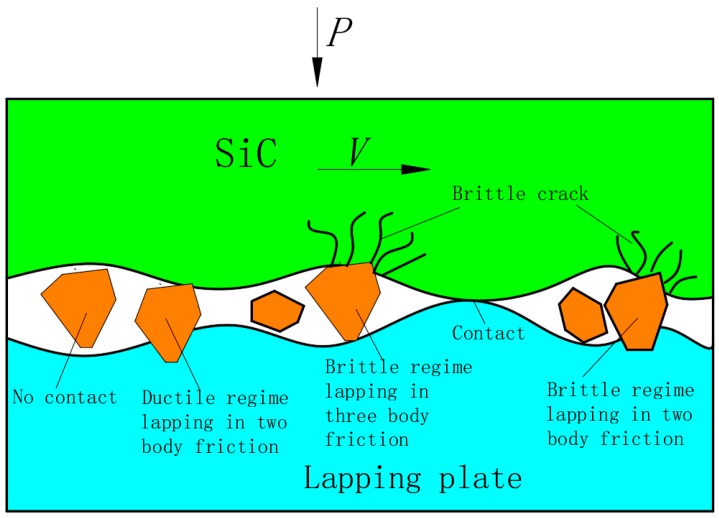
Three-Body and two-body abrasion.

Free abrasives in the form of slurry which covered the entire surface were used for lapping the workpieces. These abrasives created typical two-body and three-body friction, as shown in Figure 8. In three-body friction the abrasives are dispersed and move freely between the workpiece and the lapping plate so they simultaneously rub against the workpiece and the lapping plate; this meant that the surface materials of the workpiece are removed through surface rolling and scratching resulting in a uniform and lustrous surface consisting of countless micro broken pits. In two-body friction the abrasives become embedded into the lapping plates and only rubbed against the workpiece; this means the loading pressure is transferred directly onto the abrasives. In this process, the abrasives cut deeper into the workpiece than during three-body friction, and the workpiece materials are mainly removed through ploughing and micro-cutting modes. This indicates that the material removal rate is higher under two-body friction [29].

**Figure 9:**
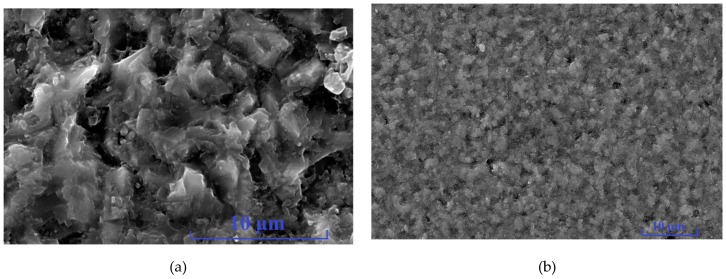
Morphology of single crystal 6H-SiC substrates after lapping. (**a**) After lapping by W14 diamond. (**b**) After lapping by W1.5 diamond.

Figure 9 shows the surface morphologies of the cast iron and ceramic plates lapped using W14 and W1.5 diamond abrasives; the surfaces of the workpieces consist of countless micro broken pits whose size depends on whether W14 or W1.5 abrasives used. This means that material was mainly removed by a larger number of brittle fractures which caused debris to break away from the material and leave many broken pits on the surface of the workpiece; this is where three-body friction occurred. The larger the diameter of the abrasives, the less abrasives per unit area and therefore a greater force was exerted on single abrasives under the same loads, and the larger the material removed, the rougher the surface was.

**Figure 10:**
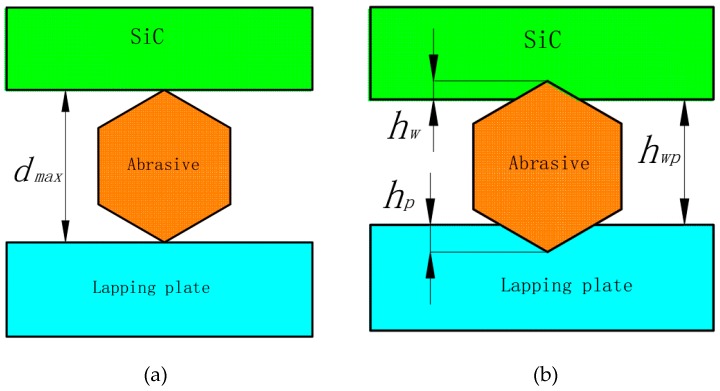
Contact state diagram of the abrasives, workpiece, and lapping plate. (**a**) Ideal contact state. (**b**) Actual contact state.

In lapping, the space $h_{wp}$ between the workpieces and the lapping plates was mainly determined by the maximum diameter $d_{\max}$ of abrasives in the space, so abrasives with diameters larger than $h_{wp}$ did most of the lapping. The distribution laws of the diameters of abrasives met the normal probability density function [30]. Suppose that abrasives with diameters larger than D50 (D50 of W14 and W1.5 was 11 μm and 1.2 μm) did most of the lapping, then abrasives filled all of the space between the workpieces and lapping plates. Based on indentation theory, the contact deformation when abrasives were pressed into the workpieces and lapping plates is shown in Figure 10. Assume that $H_{}$ and F_w_ indicate the hardness of the workpieces and the average pressure on single abrasive, then [31],

(11) $$h_{w}=\sqrt{\frac{F_{w}k_{\beta }}{H_{}}}.$$

$K_{\beta }$ is the coefficient relating to the shape of the grinding grains. Suppose that the grinding grains are ideal spheres with a radius $d_{w}$, the actual contact area between a single abrasive and the workpieces may be calculated as:

(12) $$S_{A}=\pi \left({\left(\frac{d_{w}}{2}\right)}^{2}-{\left(\frac{d_{W}}{2}-h_{W}\right)}^{2}\right).$$

In accordance with the equilibrium condition of forces, the following equation is obtained.

(13) $$pS=F_{W}K_{b}\left(4S/\pi d_{w}^{2}\right)\pi \left({\left(\frac{d_{w}}{2}\right)}^{2}-{\left(\frac{d_{W}}{2}-h_{W}\right)}^{2}\right)$$

where *p*, *S*, and $K_{b}$ show the lapping pressure, the workpiece area, and the distribution coefficient of abrasives between the workpieces and lapping plates, respectively. When the abrasives were distributed closely, the coefficient is 1. Combining Equations (11) and (13), the following equation was obtained.

(14) $$F_{W}=\sqrt{pd_{W}H_{}/4K_{b}K_{\beta }}.$$

When $K_{\beta }$ and $K_{b}$ were 1 and the nanoindentation results and grinding parameters were substituted [31], the forces of effective W14 abrasives on C and Si faces were 1723 mN and 1728 mN, separately, and the forces of effective W1.5 abrasives on C and Si faces were 569 mN and 588 mN, respectively. Obviously, where the abrasives completely filled the gaps between workpieces and lapping plates, the forces for pressing abrasives in workpieces under two process conditions were beyond the theoretical critical load for brittle fractures of the single crystal SiC substrates. However, many abrasives between the workpieces and lapping plates could not be involved in lapping, thus causing larger actual forces for pressing abrasives into workpieces. Therefore, in two process conditions such as three-body friction and rolling of abrasives, pure brittle fractures occurred on the surface of single crystal SiC, and the rolling and fragile broken accumulation removed workpiece materials and formed uniform and smooth surfaces consisting of countless micro broken pits.

### 3.4. The Cluster MR Finishing Results of Semi-fixed Abrasives and Analysis of Material Removal

In cluster MR plane finishing, small magnetic bodies are embedded into polishing plates made of diamagnetic materials according to the cluster principles. When MR fluids mixed with abrasives are poured onto the polishing plates, the upper magnetic bodies of the MR fluids are lined as chain structures along the direction of magnetic lines of forces to solidify and form semi-fixed Bingham viscoelastic micro grinding heads. This array of micro grinding heads regularly formed as polishing pads. When the workpiece is close to the polishing plates and moves along it, positive pressures and shear forces are generated in the contact areas such that the surfaces materials of the workpieces are removed. Owing to the semi-fixed and soft-constraint of viscoelastic MR fluids for the abrasives, the material on the surfaces of the workpieces are removed flexibly without damaging and scratching, so the polishing process is very efficient and results in super smooth surfaces [32].

**Figure 11:**
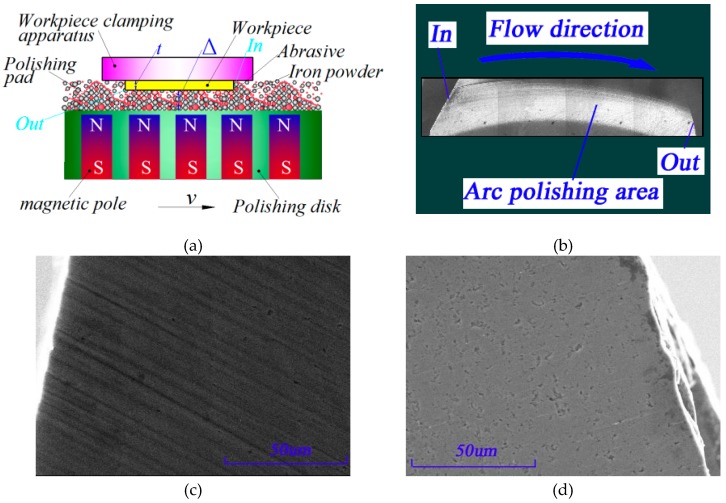
Scanning electron microscope (SEM) morphology of Single crystal 6H-SiC wafer polishing belt. (**a**) Schematic diagram of polishing, (**b**) schematic diagram of the polishing belt, (**c**) SEM morphology of entrance and (**d**) SEM morphology of exit.

Figure 11 shows the surface SEM images at the entrance and exit of the micro grinding heads in the polishing belts on the 6H-SiC substrates obtained from the experiment. Note the deep elastic-plastic grooves where the arc polishing belts entered the surfaces of single crystal 6H-SiC; these grooves were deeper at the entry edge and then became shallower towards the interior, and since there were no grooves where the arc polishing belts exited, the surfaces were smoother and flatter. However, some pits and pores generated by the lapping process remained but even though the polishing belts left deep grooves, there were no brittle removal scratches.

The removal rate of material during polishing is generally related to the mechanical properties of the polishing pads. Yong’s modulus of MR elastic polishing pads was ~1 Mpa(~2G′), which was much smaller than traditional polishing pads (~50–100 Mpa) [33]. Therefore, the abrasives constrained by magnetic connection were dislocated and deformed by the cutting forces, causing the accommodated-sinking effect. This was why the forces exerted by the abrasives onto the workpieces was small and the materials were removed from the surface of workpieces under elastic-plastic deformation. 

Furthermore, in accordance with the Preston equation, the rate of removing MR material was proportional to the pressure on the surface of workpieces and the relative speed. As Figure 11a shows, the polishing pressure *P_F_* of the MR micro grinding heads on the workpieces was a complex parameter which included hydrodynamic pressures, and pressures produced by the MR effects and liquid buoyancy. Moreover, the pressures produced by MR effects consisted of magnetizing and magnetostrictive pressures, and since MR fluids are non-compressible, the magnetostrictive pressures in the magnetic fields induced by changes in volume were approximately zero, the expression for polishing pressures is

(15) $$P_{F}=P_{d}+P_{g}+P_{m}$$

where $P_{d}$ and $P_{m}$ represent the hydrodynamic pressure and the pressure produced by MR effects of the MR fluids, respectively. Furthermore, $P_{g}$ stands for the buoyancy of MR fluids, but it was ignored in the calculation because it was much less than $P_{d}$ and $P_{m}$.

According to the studies of Feng et al. [34], the pressure produced by the MR effects of spherical magnetic particles in MR fluids due to external magnetic fields was calculated using the following equation.

(16) $$P_{m}=\frac{3\varphi \mu_{0}\left(\mu -\mu_{0}\right)}{\mu +2\mu_{0}}\int_{0}^{H_{m}}H_{m}dH_{m}$$

where $\mu_{0}$, $H_{m}$, μ and φ represent the magnetic inductivity of a vacuum, the strength of the external magnetic fields on the surface of the workpieces, the magnetic inductivity of magnetic particles, and the proportion of magnetic particles in the MR fluids, respectively. Obviously, the pressure produced by the MR effects was directly proportional to the magnetic field strength H of the external magnetic fields on the surface of the workpieces.

In cluster MR polishing, because the workpiece and the polishing plates are parallel, there is no wedge pop-in, so in theory, dynamic pressures could not be formed and there were only the pressures produced by the MR effects. However, where the micro grinding heads entered the workpieces from the bottom surface of tool heads, a pop-in of height existed, and the dynamic pressures existing at the edges of polishing belts will meet the following equation [35].

(17) $$\frac{dP_{d}}{dx}=6\eta \upsilon \frac{h-h_{0}}{h^{3}}$$

where η and $h_{0}$ denote the initial viscosity of the MR fluids and the distance (i.e., $h_{0}$ is the machining gap Δ) from the polishing plates to the surfaces of workpieces, respectively, then h represents the height from the polishing plates to the surfaces of tool heads. If the thickness of workpieces is t then h=Δ+t. 

In machining, the material removal modes are only related to the maximum force of the grinding grains, which means the forces of grinding grains in the machining gaps can be transmitted and the forces of each grinding grain and carbonyl iron powder grain are basically consistent. Moreover, the number of grinding grains and carbonyl iron powder grains in the machining gaps in the same chain is $\Delta /d_{w}$ so the abrasive forces at the entrance and the exit of the polishing belts in contact with the workpieces can be calculated approximately as:

(18) $$F_{\mathrm{in}}=\frac{\Delta }{d_{w}}\left(\frac{3\varphi \mu_{0}\left(\mu -\mu_{0}\right)}{\mu +2\mu_{0}}H_{m}+6\eta \upsilon \frac{t}{{\left(\Delta +t\right)}^{3}}\right)\;\pi {\left(\frac{d_{w}}{2}\right)}^{2}$$

(19) $$F_{\mathrm{out}}=\frac{\Delta }{d_{w}}\frac{3\varphi \mu_{0}\left(\mu -\mu_{0}\right)}{\mu +2\mu_{0}}H_{m}\;\pi {\left(\frac{d_{w}}{2}\right)}^{2}.$$

When the data in Table 3 were substituted into Equations (18) and (19), the theoretical pressures of abrasives at the entrance and exit of workpieces were computed as 1514.27 μN and 3.47 μN, respectively; this meant they were in the mechanical range for single crystal SiC producing elastic-plastic and elastic deformations and therefore single crystal SiC materials were removed in full elastic-plastic mode, thus realizing machining without any sub-surface damage. 

## 4. Conclusions

The results of nanoindentation experiments under static stiffness showed that the hardness of C and Si faces of single crystal SiC under 500 mN loads was 38.596 Gpa and 36.246 Gpa, and the elastic moduli of the C and Si faces were 563.019 Gpa and 524.839 Gpa, respectively. According to the experimental results, the theoretical critical loads for the plastic transition of C and Si faces of single crystal SiC were calculated as 1.941 mN and 1.77 mN and the theoretical loads for their brittle fractures were 366.8 mN and 488.67 mN, respectively. These results basically coincided with the experimental results of nanoindentation.

In grinding based on the rotation of workpieces using fixed abrasives, the critical grit cutting depth of abrasives on the C and Si faces of single crystal 6H-SiC were 5.3 nm and 6.1 nm, separately. When the maximum thickness of undeformed substrate relating to the processing parameters was less than the critical grit cutting depth, single crystal SiC material was removed by plastic deformation, and when the maximum thickness of undeformed substrate was larger than the critical grit cutting depth, the removal mode changed to brittle removal. Moreover, as the maximum thickness of undeformed substrate increased the proportion of brittle removal on the surfaces gradually increased.

The forces of lapping materials on C and Si faces by using free abrasives (W14) were 1723 mN and 1782 mN, while those obtained using free abrasives (W1.5) were 569 mN and 588 mN, respectively. These forces were larger than the theoretical critical loads for the brittle fractures of single crystal SiC substrates. With the three-body friction motion of abrasives, single crystal SiC material was rolled, broken, and removed under pure brittle fracture. 

In the cluster MR finishing experiments where semi-fixed abrasives were used, the theoretical pressure of abrasives at the entrance and exit of the polishing belts were 1514.27 μN and 3.47 μN, respectively. These pressures were in the range for elastic-plastic and elastic deformation of single crystal SiC. The elastic-plastic removal grooves at the entrance of the polishing belts were deeper while the surface at the exit was flatter and smoother; this coincided with the mechanical calculation and nanoindentation experiment.

This study applied the experimental results of nanoindentation to the mechanical analysis of abrasives under different fixation methods and the removal and deformation analysis of single crystal SiC material. These research results are significant for the ultra-precision machining of single crystal SiC substrates. 

## Figures and Tables

**Table 1:** Grinding conditions used in experiments.

Process No.	Grinding Wheel Type (Abrasive Grain Size)	Feed Rate *f_s_* (µm/s)	$\mathrm{Workpiece}\;\mathrm{Speed}\;n_{w}\;(\mathrm{rpm})$	$\mathrm{Wheel}\;\mathrm{Speed}\;n_{s}(\;\mathrm{rpm})$
No.1	325# (45µm)	5	151	1800
No.2	325# (45µm)	0.1	151	1800
No.3	325# (45µm)	0.1	151	3200
No.4	8000# (1.6µm)	0.1	151	3200

**Table 2:** Hardness and elastic modulus of Single crystal 6H-SiC substrates by quasi-static indentation.

	Loads	1 mN	2 mN	5 mN	10 mN	20 mN	50 mN	100 mN	200 mN	300 mN	500 mN
Hardness/ Elasticity Modulus											
Hardness of C face (Gpa)		47.867	55.418	55.825	52.596	50.514	46.745	43.741	41.435	39.861	38.596
Elasticity modulus of C face (Gpa)		538.075	581.841	627.822	618.019	612.893	596.833	576.843	567.615	562.4	563.019
Hardness of Si face (Gpa)		48.211	46.381	50.788	48.446	45.693	42.392	39.651	37.315	36.311	36.246
Elasticity modulus of Si face (Gpa)		533.033	554.146	614.533	601.814	589.13	570.998	548.221	535.773	525.946	524.839

**Table 3:** Calculating Parameters.

Parameter	Value
Thickness of workpiece *t* (mm)	0.3
Machining gap Δ (mm)	0.8
Abrasive diameter $d_{w}$ (μm)	2.8
The initial viscosity of the MR fluid η (Pa·s)	0.5
The magnetoconductivity of magnetic particles μ	2000
Vacuum permeability $\mu_{0}$	1
Magnetic field intensity *H_m_* (Gs)	2000
Speed of polishing disk *v* (m/s)	1.27
The proportion of magnetic particles in the MR fluid φ	0.33

## Nomenclature

**Symbol**	**Nomenclature**
*f**_s_***	Spindle feed(µm/s)
*n_w_*	The rotational speed of workpiece (rpm)
*n_s_*	The rotational speed of grinding wheel (rpm)
*H*	Hardness (Gpa)
*E*	Elasticity modulus (Gpa)
*K_c_*	The fracture toughness (Mpa m^1/2^)
ρ	The radius of curvature of grinding grains on the materials (mm)
*F_0_*	The contact load (mN)
*F_C_*	The contact load of C face (mN)
*F_Si_*	The contact load of Si face (mN)
*P**	The critical load for brittle materials changing from plastic deformation to brittle fracture (mN)
*P*_C_*	The critical load for C face changing from plastic deformation to brittle fracture (mN)
*φ*	The proportion of magnetic particles in the MR fluids
*h*_max_	The maximum thickness of undeformed substrate (nm)
*d_c_*	The critical grit cutting depth (nm)
*R*	The radius of the grinding wheels (mm)
*L_w_*	The circumference of layers of grinding material of the cupped grinding wheels (mm)
*C*	The concentration of grinding materials of diamond grinding wheels
*K_β_*	The coefficient relating to the shape of the grinding grains
*d_w_*	The radius of grinding grain (μm)
*r_m_*	The radius at any point on the surface of the workpieces
*v_d_*	The volume fraction of diamonds in the grinding wheel
*h_wp_*	The space between the workpieces and the lapping plates (mm)
*F_in_*	The abrasive forces at the entrance of the polishing belts (μN)
*S*	The workpiece area (mm^2^)
*P_F_*	The polishing pressures (kPa)
*P_m_*	The pressure produced by MR effects of the MR fluids (kPa)
*P_d_*	The hydrodynamic pressure (kPa)
*P_g_*	The buoyancy of MR fluids (kPa)
*μ_0_*	The magnetic inductivity of a vacuum
*μ*	The magnetic inductivity of magnetic particles
*η*	The initial viscosity of the MR fluids (Pa.s)
*v*	Speed of polishing disk (m/s)
*F_v_*	The volume fraction of the layers of material in the grinding wheels (g⁄cm^3^)
*P*_Si_*	The critical load for Si face changing from plastic deformation to brittle fracture (mN)
*K_b_*	The distribution coefficient of abrasives between the workpieces and lapping plates
*H_m_*	The strength of the external magnetic fields (Gs)
*h_0_*	The distance from the polishing plates to the surfaces of workpieces (mm)
*t*	The thickness of workpieces (mm)
Δ	The machining gap (mm)
*W*	The tooth width of layers of grinding material of the cupped grinding wheels (mm)
*F_w_*	The average pressure on single abrasive (mN)
*S_A_*	The actual contact area between a single abrasive and the workpieces (mm^2^)
*p*	The lapping pressure (kPa)
*d_g_*	The equivalent spherical diameter of diamond particles
*f*	The fraction of diamond particles that actively cut when grinding
*d*_max_	The maximum diameter of abrasives (mm)
*F*_out_	The abrasive forces at the exit of the polishing belts (μN)
